# Integrative Multi-Omics Analysis for Etiology Classification and Biomarker Discovery in Stroke: Advancing towards Precision Medicine

**DOI:** 10.3390/biology13050338

**Published:** 2024-05-13

**Authors:** Alberto Labarga, Judith Martínez-Gonzalez, Miguel Barajas

**Affiliations:** 1 Health Science Department, Public University of Navarra, 31006 Pamplona, Spain; miguel.barajas@unavarra.es; 2 Escuela Técnica Superior de Ingeniería, Universitat Rovira I Virgili, 43007 Tarragona, Spain; judith.martinez@estudiants.urv.cat

**Keywords:** ischemic stroke, methylation, mRNA, circRNA, miRNA, multi-omics, biomarkers, graph neural networks

## Abstract

**Simple Summary:**

Stroke is a devastating condition that leads to significant morbidity and mortality worldwide. To enhance our understanding of stroke pathophysiology and improve patient outcomes orldwides, it is crucial to explore high-throughput omics approaches and integrate multi-omics data. In this study, we propose a graph-based integrative approach to identify stroke-related gene expression changes using blood samples from ischemic stroke patients. Our goal is to discover biomarkers that can aid in the diagnosis, etiological classification, and management of stroke.

**Abstract:**

Recent advancements in high-throughput omics technologies have opened new avenues for investigating stroke at the molecular level and elucidating the intricate interactions among various molecular components. We present a novel approach for multi-omics data integration on knowledge graphs and have applied it to a stroke etiology classification task of 30 stroke patients through the integrative analysis of DNA methylation and mRNA, miRNA, and circRNA. This approach has demonstrated promising performance as compared to other existing single technology approaches.

## 1. Introduction

Stroke represents a substantial burden on contemporary healthcare frameworks, significantly contributing to mortality and morbidity [1]. At present, the identification of stroke largely relies on clinical assessments and imaging techniques. These methods, guided by the TOAST classification system, enable the determination of stroke etiology in approximately 75–80% of instances [2]. However, for the remaining 20–25% of stroke events, the precise causative factors remain elusive. It has been hypothesized that around 25–30% of these cases with undetermined origins might be attributed to paroxysmal atrial fibrillation (PAF) [3]. Accurately identifying the etiology of acute ischemic stroke (AIS) subtypes is crucial for guiding appropriate secondary prevention strategies. For instance, anticoagulation is recommended for cardioembolic strokes (CE), which are often linked to atrial fibrillation, while antiplatelets are advised for strokes that are due to large artery atherosclerosis (LAA). This underscores the growing necessity for biomarkers that can reliably determine the cause of stroke in a clinical setting.

Biomarkers serve as objective measures for evaluating normal or pathological states, monitoring therapeutic responses, and forecasting clinical outcomes. These include diverse biological entities such as proteins, ribonucleic acids (RNAs), lipids, and metabolites.

Recent research has focused on the application of different technologies to characterize the composition of thrombi, even at the single-cell level [4,5,6,7], and many studies have tried to identify genetic biomarkers in blood both for onset and outcome predictions [8,9,10].

The techniques employed include, but are not limited to, proteomics, metabolomics, and transcriptomics, which are often applied in combination. While a multitude of statistical methods have been formulated to independently analyze large-scale, high-quality omics data, these methods, focusing on singular omics levels, often fail to consider the interplay among various molecular entities. This isolated approach risks overlooking biologically pertinent information. As a result, the translation of these biomarkers into clinical practice is hindered by their low sensitivities and specificities, and there currently exists no biomarker that simultaneously meets the criteria of high sensitivity, specificity, rapidity, accuracy, and cost-effectiveness for routine stroke management.

The integrated analysis that combines data from diverse omics approaches is increasingly recognized as vital [11]. The integration of this multi-omics data enables a comprehensive view across various biological levels, significantly enhancing our comprehension of the underlying biological mechanisms at play, and it has started being applied to stroke research [12].

Several common strategies are typically employed for multi-omics data integration and analysis:

- Correlation Analysis: This approach involves assessing the pairwise correlations between omics datasets. By examining the co-variation patterns between different types of omics data, researchers can identify relationships and potential regulatory mechanisms.
- Dimensionality Reduction Techniques: Dimensionality reduction techniques, such as principal component analysis (PCA) and independent component analysis (ICA), are used to reduce the high-dimensional nature of omics data. These techniques extract relevant features and capture the major sources of variation within the data, facilitating data integration and visualization.
- Integrative Clustering: Integrative clustering methods aim to identify clusters or subgroups of samples based on the integration of multi-omics data. These techniques consider similarities and dissimilarities across different omics layers, enabling the identification of distinct molecular subtypes or phenotypes.

More recently, autoencoders [13] have been utilized for multi-omics data integration by leveraging their ability to learn a compressed representation or latent space of the input data. Autoencoders are neural network architectures consisting of an encoder and a decoder. The encoder maps the input data to a lower-dimensional latent space, while the decoder reconstructs the original input data from the latent representation. Some examples of models in the multi-omics data integration include AIME [14], MAE [15], and many others [16].

Machine learning on graphs is also becoming an ubiquitous task in biology and biomedicine, with applications ranging from function prediction to drug repurposing, and knowledge graphs play a key role as sources of graph-structured data [17,18,19]. As such, a knowledge-graph-based integration approach with multi-omics data makes it amenable to being easily exploited by deep learning models such as Graph Neural Networks (GNNs) [20] by leveraging the graph structure inherent in the data. GNNs are a type of neural network specifically designed to process and analyze graph-structured data, such as biological networks or interaction networks in multi-omics contexts. Traditional neural networks, which expect fixed-size inputs and lack mechanisms to handle the permutation invariance and complex relational information present in graphs, are ill-equipped for such tasks. GNNs address these challenges by incorporating the connectivity patterns and features of nodes into their computational process, allowing them to learn representations for nodes, edges, or entire graphs. A pivotal aspect of GNNs is their ability to propagate and transform node features through the graph structure, enabling the capture of topological characteristics of the data.

Graph Convolutional Networks (GCNs) [21] are a prominent category within GNNs that generalize the concept of convolution from grid-like data to graphs. The convolutional operation in GCNs is generally simplified to directly aggregate and transform neighbor features, reducing computational complexity and making the model more scalable. These models have been applied successfully in node classification tasks, link prediction, and graph classification, showcasing their ability to harness both local and global graph structures for informed decision-making in a myriad of applications [22].

Taking into account the fact that biological data can be easily represented as a network where nodes represent the different biological components (genes, proteins, etc.) and edges represent the relationship between them, enabling machine learning to incorporate information about the structure of multi-omics knowledge graphs into the model opens new avenues to make predictions or discover new patterns using this relational knowledge for application in new use cases [23,24,25]. For example, a graph classification task, which predicts an attribute of each graph in a collection of graphs, can be used for patient classification. In the same way, node classification tasks predict an attribute of each node in a graph, which enable them to be used for biomarker discovery. Another interesting use case is the use of the node feature prediction task for data imputation. Finally, link prediction to predict an attribute of edges in a graph can be used to predict whether an edge should exist in the graph with application on associations of bioentities such as miRNA-target.

In our study, we have comprehensively profiled 30 acute stroke patients with different etiologies defined by TOAST classification using both transcriptomic (mRNA, miRNA, and circRNA) and epigenomic (DNA methylation) techniques in order to perform a graph-based, multi-omics integrative analysis that holds the potential to yield a more nuanced understanding of the processes involved and help in the identification of etiology-associated biomarkers that can be easily measured in blood after stroke to guide treatment strategies.

The joint analysis of methylation and mRNA, circRNA, and microRNA expression presents a comprehensive approach to understanding the intricate regulatory networks that govern cellular processes. DNA methylation serves as a pivotal epigenetic mechanism, influencing gene expression without altering the underlying DNA sequence. Methylation typically occurs in the cytosine bases in DNA, particularly in cytosine-phosphate-guanine (CpG) dinucleotides. In the human genome, CpG sites are often clustered in regions called CpG islands, which are frequently located near or within gene promoters. When methylation occurs in the promoter region of a gene, it usually leads to the suppression or silencing of that gene. This happens because the methyl groups added to the DNA can physically impede the binding of transcription factors necessary for gene expression. By analyzing methylation patterns alongside mRNA profiles, we can elucidate the epigenetic modifications that drive or suppress gene expression, offering insights into the underlying mechanisms of disease [26,27]. Meanwhile, the integration of circRNA and miRNA expression data adds a further layer of complexity and regulatory control. MicroRNAs (miRNA) are small, non-coding RNA molecules that can target multiple mRNAs and play a critical role in the regulation of gene expression and gene silencing at the post-transcriptional level [28]. Circular RNAs, (circRNA) are single-stranded RNA molecules of endogenous origin that form a circular structure through covalent bonding [29]. Notably, these molecules are evolutionarily conserved and are present in large quantities in the human transcriptome [30]. CircRNAs are recognized for their diverse regulatory roles in RNA biology and gene expression. An example of their functions includes acting as sponges to absorb microRNAs or RNA-binding proteins, thereby influencing the expression of specific target genes [31]. By exploring the dynamic interplay between these molecular entities, we can uncover regulatory networks that are critical in stroke pathogenesis and progression.

We propose the construction of a graph neural network (GNN) that can incorporate these different omics layers and exploit the existing biological databases to represent relationships or interactions between them. We can then apply graph convolutional operations to propagate expression information through the graph structure. Graph Convolutional Networks (GCNs) leverage the graph topology to update the node features based on their neighborhood relationships. This enables capturing local and global patterns within the multi-omics data and allows downstream analysis such as patient classification or biomarker discovery. This novel model aims to overcome the challenges faced by previous approaches and provide improved prediction capabilities for our etiology classification and biomarker discovery tasks.

## 2. Materials and Methods

### 2.1. Study Population

Patients presenting acute ischemic stroke were enrolled from the emergency department of the Hospital of Navarra, provided that they arrived within 4.5 h of symptom onset. Blood samples were collected from these patients within the first 24 h following admission. Informed consent was obtained from all participants, and the study was approved by the local ethics committee under project number 2015/21. From a total cohort of 700 patients recruited between January 2015 and December 2016, 30 were selected for the discovery cohort analyzed in this study. Another 50 patients were identified as a validation cohort. To ascertain the etiology of each stroke, a series of diagnostic tests were conducted. These included an electrocardiogram (EKG), chest radiography, a complete blood count, a blood biochemistry analysis, carotid ultrasonography, a transcranial Doppler (TCD) examination, non-contrast cranial tomography (CT) at baseline, an echocardiogram, and 24-h Holter monitoring. Based on the findings from these tests, patients were categorized into etiological subgroups in accordance with the Trial of ORG 10,172 in Acute Stroke Treatment (TOAST) criteria [2].

### 2.2. mRNA Expression

For gene expression profiling, total RNA was extracted using the miRNeasy Mini kit (Qiagen, Hilden, Germany) and labeled using Agilent’s Quick Amp Labeling Kit. Microarray analysis was performed using Agilent SurePrint G3 Human Gene Expression 8 × 60 K v3. Post-hybridization, the microarrays were scanned using the Agilent Technologies G4900DA SG12494263 scanner. For data processing and analysis, we employed Agilent Feature Extraction software, version 11.0.1.1. We followed manufacturer’s protocol to ensure the precise and reliable acquisition of gene expression data for our analysis. Five samples failed quality control, and their correlation with other samples was not good, so they were removed from analysis.

### 2.3. MicroRNA Expression

To assess the miRNAs levels in blood after stroke, we processed the samples as follows: samples were labeled using the miRCURY LNA™ microRNA Hi-Power Labeling Kit, Hy3™/Hy5™ and hybridized on the miRCURY LNA™ microRNA Array (7th Gen), following a single-color experimental design.

The miRCURY 7th generation array of our array contains 3100 capture probes, covering human, mouse, and rat microRNAs annotated in miRBase 19.0, as well as all viral microRNAs related to these species. In addition, this array contains capture probes for 25 miRPlus™ human microRNAs. In total 1919 human microRNAs are targeted by the platform.

To measure miRNAs levels in blood after stroke we made use of the miRCURY LNA™ microRNA Array. This array, which covers both human, mouse and rat microRNAs annotated in miRBase 19.0, targets 1919 human microRNAs. Blood samples were labeled using the miRCURY LNA™ microRNA Hi-Power Labeling Kit Hy3™/Hy5™ and hybridized onto the array following a single-color experimental design. The array slides were scanned using the Agilent G2565BA Microarray Scanner System (Agilent Technologies, Inc., Santa Clara, CA, USA) and standard image analysis to extract background corrected and normalized data was carried out using the ImaGene 9.0 software (Biodiscovery Inc., El Segundo, CA, USA).

### 2.4. Microarray Expression of circRNAs

For the circular RNA detection, the total RNAs were digested with RNase R (Epicentre, Inc., Lindenhurst, IL, USA) to remove linear RNAs and enrich circular RNAs. Then, the enriched circular RNAs were amplified and transcribed into fluorescence-labeling complementary RNA (cRNA), utilizing a random priming method (Arraystar Super RNA Labeling Kit, Arraystar, Rockville, MD, USA). The labeled cRNAs were purified by RNeasy Mini Kit (Qiagen) and hybridized onto the Arraystar Human circRNA Array V2 (8 × 15 K, Arraystar). After hybridization and washing, the arrays were scanned by the Agilent Scanner G2505C (Agilent Technologies, Inc., Santa Clara, CA, USA). Scanned images were processed using Agilent Feature Extraction software (version 11.0.1.1) for the extraction of raw data [17].

### 2.5. Genome-Wide DNA Methylation Profiling

CpG methylation levels were profiled genome-wide by using the Infinium HumanMethylationEPIC BeadChip array (Illumina, Inc., San Diego, CA, USA). Following the manufacturer’s protocol, 500 ng of genomic DNA from each blood sample was isolated by the standardized salting-out method and then bisulfite treated and hybridized to the BeadChip. Microarray image processing was carried out using the Genome Studio Methylation Module (v1.8.5).

Following standard practice for methylation data analysis [32], probes that overlap common single nucleotide polymorphisms (SNPs), as well as those annotated as internal controls, were removed. Probes located on the X and Y chromosomes, along with those previously described to hybridize to multiple locations in the genome, were also discarded [33,34]. Additionally, probes not passing Illumina quality thresholds (bead count < 3 in >5% of samples and 1% of samples with a detection *p* value > 0.05) were filtered. Finally, background correction and type I/II assay chemistry bias adjustment were applied.

### 2.6. Annotation and Biomedical Knowledge Graph Construction

Gene Expression Omnibus (GEO) annotations were used to map probe array identifiers to corresponding gene symbols, sequences, or genomic coordinates. Table 1 reflects the platform definition used.

Probe identifiers for the HumanMethylationEPIC and Human Gene Expression arrays were mapped to Ensembl Ids [35]. miRBase [36] and miRTarBase [37] were also downloaded to generate the association matrix between the microRNAs and their corresponding gene targets. The Circinteractome [38] tool provided a list of miRNAs that were potentially targeted by the analyzed circRNA. These data were used to build the association matrices between the different omics technologies and can be viewed as a biomedical knowledge graph that could be further extended with existing interaction databases such as Reactome [39].

### 2.7. Graph-Based Multi-Omics Data Integration

The proposed approach aims to predict stroke etiology by simultaneously training multiple heterogeneous networks using an extended GraphSAGE [40] model.

Graph Neural Networks (GNNs) leverage a message-passing mechanism to generate a representation of a node aggregating information from its neighbors up to a hop distance. While traditional GCN models typically rely on the entire graph for training, a key characteristic of GraphSAGE is that it does not necessitate the entire graph structure to be present during the learning process because it samples a fixed number of neighbors for aggregation. This feature allows GraphSAGE to effectively handle large graphs by learning from a sample of the nodes, and it can be used to generate representations of new nodes. While GraphSAGE was designed to work with a single graph, some attempts have been performed to extend it to two interconnected graphs [41].

Typically, an undirected graph *G* = (*V*, *E*) with *n* nodes and *m* edges is represented using an adjacency matrix denoted by *A*∈{0, 1}*n*×*n*, with each element *Aij* = 1 if there exists an edge between node *vi* and *vj*, otherwise *Aij* = 0. Each node is associated with a *d*-dimensional feature vector, and the feature matrix for all nodes is represented as $X\in R^{n\times d}$.

In our case, we first add the nodes relative to each of the omics analyzed and create links between then according to the information of the knowledge graph, and the patient nodes, and initialize their corresponding embeddings randomly. The embedding size chosen is 100. The expression matrix for each of the omics serves as relationship measure between the patient and the corresponding biological nodes.

The calculation of the node ***u*** embeddings at layer *k* consists in the embeddings of these chosen neighbors, synthesizing an updated representation for the focal node ***u***. Using mean aggregation function, this can be expressed as

(1) $$h_{u}^{\left(k\right)}=\sigma \left(W\cdot MEAN\left(\left\{h_{u}^{\left(k-1\right)}\right\}\cup \left\{h_{u}^{\left(k-1\right)},\forall v\in N(u)\right\}\right)\right)$$

where ***σ*(.)** is the non-linear activation function, ***W*** refers to the weights matrix, ***v*** are the neighbor nodes, and ***h*** the node embeddings.

Relevant neighbor selection is particularly important in our multiple layer architecture. Graph Attention Network (GAT) [42] brings the well-known attention mechanism behind the Transformer architecture [43] to the realm of graph neural networks. Our model assigns varying levels of importance to nodes in a neighborhood, which allows it to focus on the most relevant parts of the graph structure for the task at hand. This can be expressed as

(2) $$h_{u}^{k}=\sigma \left(\sum_{{v\in N(u)}^{\alpha_{vu}W^{k}h_{v}^{k-1}}}\right)$$

where

(3) $$\alpha_{uv}=\frac{1}{\left|N(u)\right|}$$

refers to the weighing factor determining the importance of the message of node ***v*** to node ***u***.

**Figure 1:**
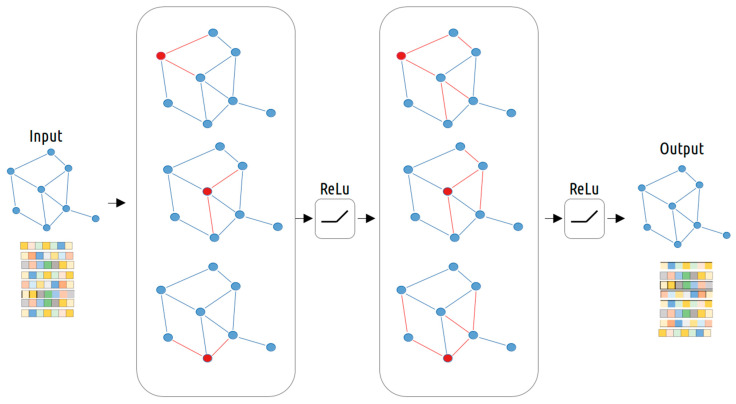
Illustration of GraphSAGE model with a sampling strategy. Red denotes target nodes that need to aggregate information from neighbors. The red lines denote the information stream for first-hop and second-hop neighbors, respectively. As a result, the output contains both first-hop and second-hop information. Here, the maximum number of neighbors for each hop is set as two.

These networks are then merged into a multi-layer network for a two-step training process. Figure 1 illustrates this process for the two-layer GCN architecture used by our system. First, a fixed number of nodes is selected across the different graphs and the aggregation function is applied, first for one-hope neighbors, and then for two-hop neighbors.

Finally, we apply a softmax classification layer to the unified patient embeddings to classify patients into the predefined etiology subtypes. The input to this layer is the fused patient embedding, while the output is a probability distribution over the possible classes.

During training, we optimized the model using cross-entropy loss. The model was trained end-to-end, allowing the GraphSAGE-based aggregation functions, the cross-graph feature enhancement, the embedding fusion, and the patient classification layer to all adapt based on the backpropagation of the loss.

**Figure 2:**
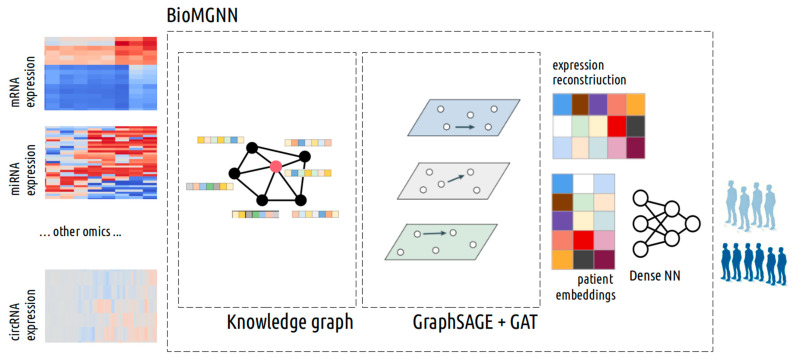
BioMGNN workflow. The original expressions (circRNA. miRNA, etc.) are projected into a knowledge graph that captures the known biological relationship between the different features together with the patient nodes to create a multipartite network. Random embeddings are generated for each nodes, and the GraphSAGE with the attention algorithm proposed is applied. After convergence, the final patient embeddings are used to predict the stroke etiology.

This results in the generation of a collection of embeddings for the different omics and for the patients. These patient embeddings serve as the final feature representations for predicting stroke etiology. The complete process, which we named the Biological Multilayer Graph Neural Network (BioMGNN), is illustrated in Figure 2.

## 3. Results

### 3.1. Patient Characterisation

For each participant, a comprehensive record of vascular risk factors was compiled, including hypertension, atrial fibrillation, diabetes mellitus, dyslipidemia, tobacco use, cardiovascular disease, and peripheral atherosclerosis. The demographic and clinical characteristics of patients, classified by stroke etiology, are summarized in Table 2.

### 3.2. Patient Classification Task

We implemented the proposed BioMGNN model using PyTorch Geometric (version 2.1.0) [44], a Python library built upon PyTorch [45]. We used an Adam optimizer [46] to train the model on a single NVIDIA H100 GPUs with 80 Gb of memory. After training, the final patient embeddings were obtained.

We have compared the performance of our algorithm with the performance of the individual data to predict the patient’s etiology. For this, we used the Xgboost classifier [37] on the original individual datasets. The average classification evaluation metrics results using five-fold cross-validation are presented in Table 3.

**Figure 3:**
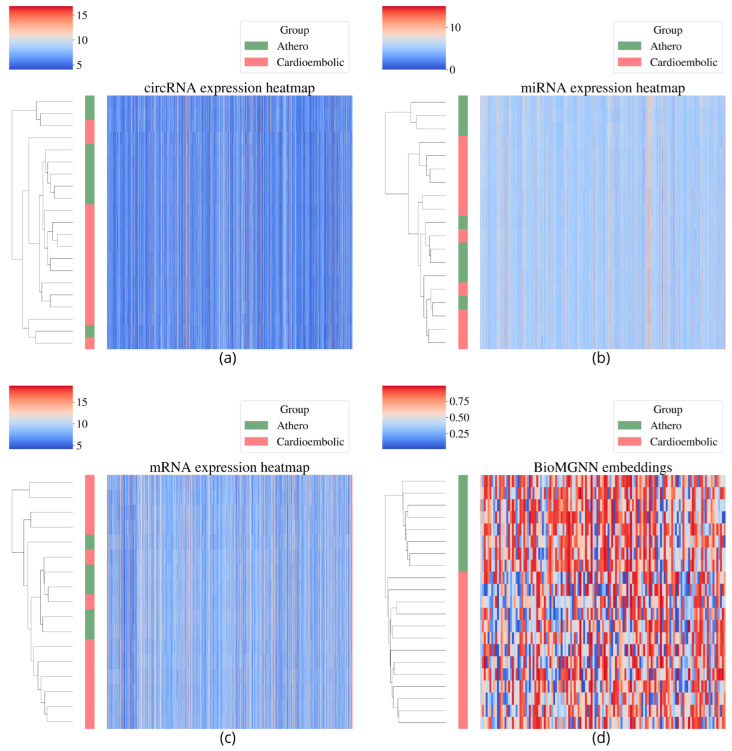
Visual exploration of the expression data using a clustered heatmap: (**a**) Expression levels of the original circRNA data, (**b**) expression levels of the original miRNA data, (**c**) expression levels of the original mRNA data, and (**d**) bioMGNN-calculated patient embeddings. The green (Cardioembolic) and blue (Atherosclerotic) left bar indicates patient etiology. BioMGNN shows better performance in clustering similar patients than the original data sources.

The BioMGNN-generated embeddings performed much better than the individual technologies original data, even after feature selection. This can also be seen in Figure 3 where clustering of the used data is shown.

### 3.3. Biomarker Module Discovery

In the context of our study, attention weights are learned for node *I* in a network and its neighbor node *j*, and it can be interpreted as the probability of how much impact the node *j* has in learning the representation of node *i*.

The most relevant node interactions for differentiating between stroke subtypes are identified through the attention weight matrix associated with the nodes. In this matrix, the magnitude of the attention weight is directly proportional to the significance of a node pair in differentiating subtypes. Our model learns these attention weights by assessing the relative importance of neighboring genes for each gene in the network.

To extrapolate global patterns from these attention weights across node pairs, we amplify the attention weights by the degree of the nodes, that is, the number of nodes it connects to.

Establishing a suitable threshold, we can select multiple node pairs to form a comprehensive set of biomarkers across the different layers, offering insights into the complex interplay of molecules relevant to the stroke subtypes in question.

**Figure 4:**
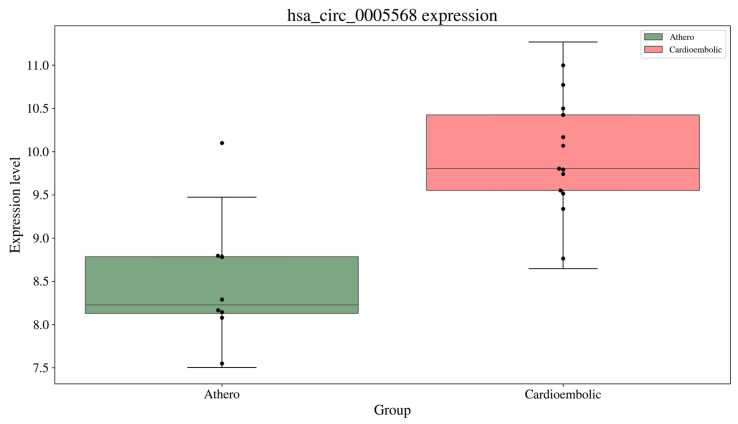
Expression levels of hsa_circ_0005568 in Atherosclerotic vs. Cardioembolic groups. These differential expression levels were further confirmed by RT-qPCR in a validation cohort.

We carried out some functional analysis on some of these selected sets using gene set enrichment analysis (GSEA) methodology [47]. One of the sets resulted in Myc targets-related biomolecules. It has been shown that the elevation of c-myc or the suppression of miR-200b-5p improved neurological function, reduced inflammation and neuronal apoptosis, and attenuated brain tissue pathology and neuronal survival of the middle cerebral artery occlusion (MCAO) mouse model [48]. Another set included circRNA hsa_circ_0005568 and some miRNAs enriched in pathways such as lysine degradation, fatty acids biogenesis, and arrhythmogenic right ventricular cardiomyopathy (ARVC). Significant expression level differences for the genes THBS3 and AMIGO2 were also detected by BioMGNN as part of this biomarker module. Thrombospondin 3 (THBS3) is part of the thrombospondin family, which is involved in cell-to-cell and cell-to-matrix communication. It plays crucial roles in tissue remodeling and angiogenesis, which the development of new blood vessels. These processes are critical in post-stroke recovery and in cardiovascular diseases where tissue repair and angiogenesis are needed. AMIGO2 is involved in cell adhesion and signaling. It can influence neuronal maturation and may play roles in neural circuit formation and recovery mechanisms post-stroke. hsa_circ_0005568 differential expression levels (Figure 4) were confirmed in the validation cohort using RT-qPCR, making it an interesting candidate worth exploring in future studies [49]. The expression levels of the module components are presented in Supplementary Figure S1.

### 3.4. Comparison with Other Biomarker Discovery Methodologies

The effectiveness of the biomarkers identified through our study was assessed based on their predictive accuracy in classifying subtypes using unseen data.

We benchmarked the performance of our method against two established multi-omics biomarker discovery tools: MOFA (Multi-Omics Factor Analysis) [50] and MOGONET (Multi-Omics Graph cOnvolutional NETworks) [51].

Xgboost was employed as the classification model to evaluate the predictive efficacy of the identified biomarkers using a 10-fold cross-validation (CV) strategy aimed to ensure a comprehensive and rigorous evaluation of our biomarkers’ validity in subtype classification tasks. We split the dataset in 10 groups. Then, each group was selected as the test set to evaluate the performance of a model trained on the other groups.

The performance of our predictive model was quantitatively evaluated using the area under the receiver operating characteristic curve (AUC) for its effectiveness in measuring the accuracy and reliability of classification models, particularly in biomedical applications where the cost of false positives and false negatives can be high.

**Figure 5:**
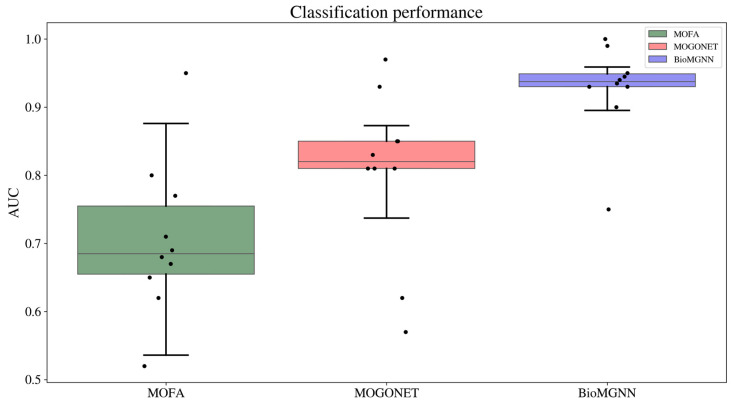
Classification performance (AUC) comparison for the selected biomarker discovery methods using a 10-fold cross-validation (CV) strategy on the patient etiology classification task using Xgboost classifier.

The results, shown in Figure 5, suggests that our method has a robust predictive capability, potentially offering enhanced accuracy over existing tools in the context of multi-omics biomarker discovery. The ability of BioGMGNN to consistently outperform in subtype prediction, regardless of the train-test set configurations, underscores its effectiveness and reliability as a tool in the field of precision medicine and biomarker discovery.

For those interested in a more detailed understanding of our benchmarking scheme and the specifics of hyperparameter tuning for the different methods, the code to reproduce the analysis is available at the ICTUSENSOPT project’s Github repository https://github.com/alabarga/ictusensopt/tree/main/benchmark (accessed on 20 March 2024).

## 4. Discussion

A common approach in multi-omics data analysis involves transforming the data into a unified feature space, creating latent representations of the samples. Key methods in this domain include matrix factorization [52], sparse-generalized canonical correlation analysis (sGCCA) [53], and multi-omics factor analysis (MOFA). While these techniques were not originally conceived for the purpose of multi-omics biomarker discovery, they offer a significant advantage. The latent representations of patients they generate can be incorporated into classification models, making these methods adaptable for biomarker identification.

Certain multi-omics integration methods have been expressly developed for biomarker discovery, exemplifying a focused approach in this field. The Data Integration Analysis for Biomarker discovery using Latent cOmponents (DIABLO) [54] is a notable instance. DIABLO employs sparse Generalized Canonical Correlation Analysis (sGCCA) to integrate multi-omics data, effectively capturing the common biological variations across different omics by maximizing their intercorrelation. While these methods consider the relationships among various omics types, they are not specifically tailored to identify gene-level biomarkers, which necessitates considering the complex interactions among biomolecules.

On the other hand, the Multi-Omics Graph cOnvolutional NETworks (MOGONET) represents an interesting graph-based approach in this domain. MOGONET learns omics-specific features from graphs built on patient-specific similarity matrices for each omic technology for later integration through a view correlation discovery network. This process aids in uncovering latent cross-omics correlations. However, a limitation of MOGONET is that its integration process is focused on the patient level, thereby potentially overlooking the intricate interplay between different omics features. This highlights a gap in current methodologies, pointing to the need for more nuanced models that can capture the detailed interactions within multi-omics data.

Another recent work proposes the multi-omics, gene-centric biomarker discovery framework Graph Attention Networks [55]. They used a two-step procedure where a Random Walk biomarker prioritization using differentially expressed proteome or metabolome anchors as the origin of the flow of information and then a graph transformer is used to model the relations between the selected genes to generate patient embeddings in a patient classification task. Biomarker genes are the selected using the attention weights in a similar way than our BioMGNN approach. However, this approach is based on the gene-based features that represent multi-omics data without being able to capture causal effects from the regulatory network as BioMGNN does.

In this paper, we propose an end-to-end, supervised multi-omics integration method named BioMGNN for biomedical classification tasks, which learns the patient similarity network that is beneficial to classification tasks while selecting important biomarkers. This multi-omics representation learning can effectively capture complex common and complementary information between omics during multi-omics integration. In addition, weighting the embedding representations of different omics through the multi-omics attention mechanism can improve classification performance and can also be used to efficiently identify meaningful potential biomarkers using the learned embeddings and attention weights. Our initial assessment suggests that BioMGNN stands out as a promising tool for these tasks.

Moving forward, there are several areas of potential future work that are needed to enhance our multi-omics data analysis platform. The first involves exploring further graph network architectures and optimizing training performance. Additionally, expanding ontology mapping to cover more domains and integrating external data sources would increase the scope of our standardization efforts. Validating and evaluating results against gold-standard multi-omics datasets, involving domain experts, and developing a user-friendly library for researchers to run their own analysis are crucial next steps. These future endeavors will refine and advance our methodology, increasing its impact and adoption in bioinformatics.

In a discovery study like ours in which a large number of molecules is analyzed in a relatively small group of subjects, one clear limitation is the limited sample size. This factor makes us cautious in drawing conclusions. To ensure the external validity of our findings, it is essential that they be replicated in the validation cohort of ischemic stroke patients. This validation must include more diverse study populations, as a lack of ethnic and geographical diversity among participants in our study is currently an important bias.

## 5. Conclusions

We have successfully showcased the effectiveness of the Multi-layer Graph Convolutional Network approach in extracting pertinent information from multi-omics expression data, particularly in regard to tackling the stroke etiology classification challenge. The knowledge-driven simultaneous analysis of multiple molecular levels exposed networks of interactions that can be further explored as stroke etiology biomarkers which could hardly be discovered by the individual data analysis.

The performance of BioMGNN in these initial stages indicates its potential utility in the field, emerging as an end-to-end, interpretable multi-omics integration method, although further validation with larger and more diverse cohorts is required to fully establish its efficacy and applicability.

We believe that the development of novel techniques that make use of the latest advances in artificial intelligence and foundation models research, together with a much more complete study population in terms of the study variables and diversity, will help to improve the prediction capacity of our model. This would allow the development of a biomarker discovery framework with broad application in both personalized medicine and treatment decision-making in the near future.

## Figures and Tables

**Table 1:** Gene Expression Omnibus platforms for the array used in this study.

	GEO Platform	Array Name
circRNA	GPL21825	Arraystar Human CircRNA microarray V2
methylation	GPL21145	Infinium MethylationEPIC
microRNA	GPL19322	miRCURY LNA microRNA Array, 7th gen
mRNA	GPL21185	Agilent-072363 SurePrint G3 Human GE v3 8 × 60 K

**Table 2:** Demographic and clinical characteristics of the patients included in the study.

	Atherothrombotic (*n* = 8)	Cardioembolic (*n* = 14)	Undetermined (*n* = 8)
Age—years, median (IQR)	70 (55–80)	75 (70.5–77)	66.5 (49–77)
Male, *n* (%)	7 (87.5)	7 (50)	5 (62.5)
High blood pressure, *n* (%)	6 (75)	12 (85.7)	3 (37.5)
Diabetes mellitus, *n* (%)	2 (25)	2 (14.3)	2 (25)
Dyslipidemia, *n* (%)	3 (37.5)	9 (64.3)	5 (62.5)
Smoker, *n* (%)	4 (50)	2 (20)	3 (42.9)
Cardiopathy, *n* (%)	3 (37.5)	6 (42.9)	0 (0)
Atrial fibrillation, *n* (%)	0 (0)	15 (100)	0 (0)
Peripheral arteropathy, *n* (%)	2 (25)	0 (0)	0 (0)
Basal mRankin, median (IQR)	0.5 (0–1)	0 (0–1.25)	0 (0–0.75)
Basal NIHSS, median (RIQ)	8.5 (5–18)	20 (17–22)	19 (18–20)
Significant ipsilateral carotid stenosis (%)	8 (100)	0 (0)	1 (14.3)
Hemorrhagic transformation, *n* (%)	5 (62.5)	4 (28.6)	2 (25)
Discharge mRankin, median (IQR)	4.5 (2–6)	4 (2–5)	3 (0.5–5)

**Table 3:** Evaluation metrics for the patient etiology classification task.

	BioMGNN	miRNA	circRNA	Methyl	mRNA
accuracy	0.95	0.48	0.52	0.67	0.77
precision	0.93	0.43	0.55	0.65	0.77
recall	0.95	0.48	0.52	0.67	0.77
F1 score	0.96	0.50	0.54	0.78	0.86
AUC	0.95	0.40	0.58	0.60	0.90

## Data Availability

The raw datasets used and/or analysed during the current study are available from the corresponding author on reasonable request. Example datasets and code to reproduce the biomarker analysis can be found at https://github.com/alabarga/BioMGNN (accessed on 20 March 2024).

## Supplementary Materials

The following supporting information can be downloaded at: https://www.mdpi.com/article/10.3390/biology13050338/s1, Figure S1: Expression levels of hsa_circ_0005568 module components in Atherosclerotic vs. Cardioembolic groups; Table S1: miRNA and circRNA biomarker modules detected.
