# Supplementary material for: Integrative Multi-Omics Analysis for Etiology Classification and Biomarker Discovery in Stroke: Advancing towards Precision Medicine
Source: Biology (Basel). 2024 May 13;13(5):338. doi: 10.3390/biology13050338 (PMC11149453; doi:10.3390/biology13050338)
Supplement: Supplementary file 1 [file biology-13-00338-s001.zip › supp_figures.pdf]

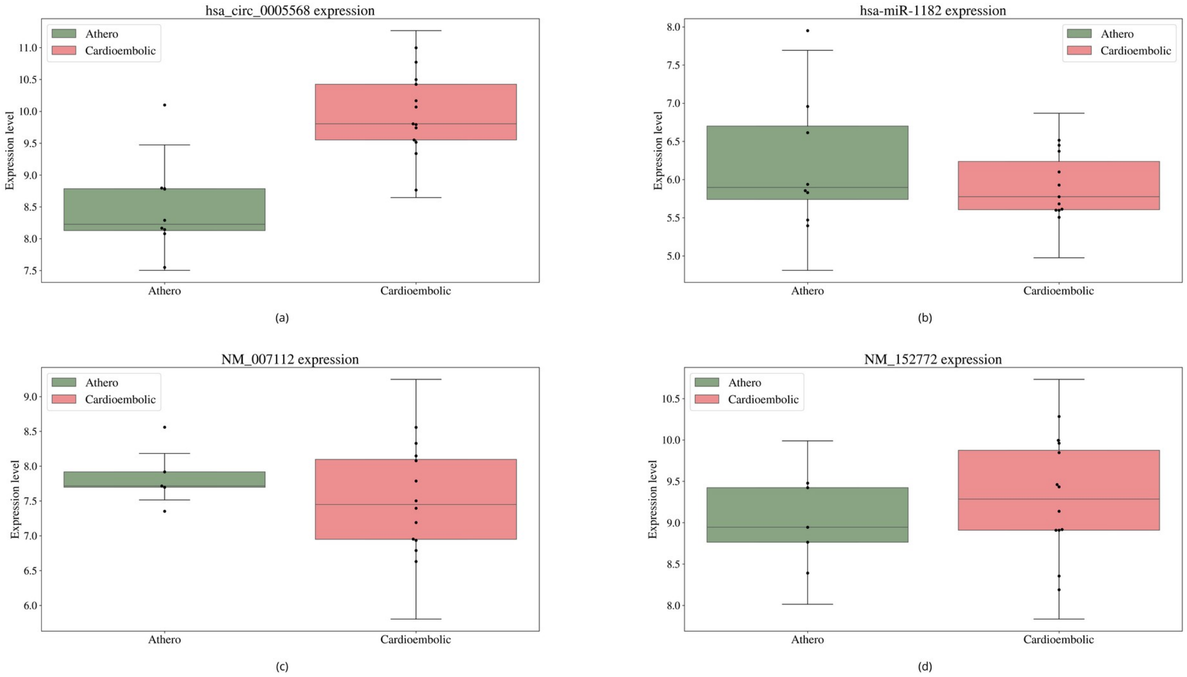

Supplementary Figure S1. Expression levels of the features present in the same module than circRNA hsa_circ_0005568. Thrombospondin 3 (NM_007112) is part of the thrombospondin family, which are involved in cell-to-cell and cell-to-matrix communication. They play crucial roles in tissue remodeling and angiogenesis, the development of new blood vessels. These processes are critical in post-stroke recovery and in cardiovascular diseases where tissue repair and angiogenesis are needed. AMIGO2 (NM_152772) is involved in cell adhesion and signaling. It can influence neuronal maturation and may play roles in neural circuit formation and recovery mechanisms post-stroke.
